# Identification and validation of SLCO4C1 as a biological marker in hepatocellular carcinoma based on anoikis classification features

**DOI:** 10.18632/aging.205438

**Published:** 2024-01-15

**Authors:** Tianbing Wang, Kai Guo, Shoushan Yang, Di Zhang, Haodong Cui, Jimin Yin, Shuhui Yuan, Yong Wang, Yong Qi, Wenyong Wu

**Affiliations:** 1 Department of General Surgery, Anhui No. 2 Provincial People’s Hospital, Hefei 230000, China; 2 Anhui No. 2 Provincial People’s Hospital Clinical College of Anhui Medical University, Hefei 230000, China; 3 Anhui No. 2 Provincial People’s Hospital, Hefei 230000, China; 4 The Fifth Clinical Medical College of Anhui Medical University, Hefei 230000, China; 5 Department of General Surgery, Luan Fourth People’s Hospital, Luan 237000, China; 6 Clinical Genomic Center, Hefei KingMed for Clinical Laboratory, Hefei 230000, China; 7 Anhui Huaheng Biotechnology Co., Ltd., Hefei 230000, China; 8 Department of General Surgery, The Second Affiliated Hospital of Anhui Medical University, Hefei 230000, China

**Keywords:** hepatocellular carcinoma, anoikis, prognostic biomarker, immune cells infiltration, tumor microenvironment, drug sensitivity, SLCO4C1

## Abstract

Background: Hepatocellular carcinoma (HCC) exhibits a high degree of invasiveness and is closely associated with rapid disease progression. Multiple lines of evidence indicate a strong correlation between anoikis resistance and tumor progression, invasion, and metastasis. Nevertheless, the classification of anoikis in HCC and the investigation of novel biological target mechanisms in this context continue to pose challenges, requiring further exploration.

Methods: Combined with HCC samples from TCGA, GEO and ICGC databases, cluster analysis was conducted on anoikis genes, revealing novel patterns among different subtypes. Significant gene analysis of different gene subtypes was performed using WCGNA. The anoikis prognostic risk model was established by Lasso-Cox. Go, KEGG, and GSEA were applied to investigate pathway enrichment primarily observed in risk groups. We compared the disparities in immune infiltration, TMB, tumor microenvironment (TME), and drug sensitivity between the two risk groups. RT-qPCR and Western blotting were performed to validate the expression levels of SLCO4C1 in HCC. The biological functions of SLCO4C1 in HCC cells were assessed through various experiments, including CCK8 assay, colony formation assay, invasion migration assay, wound healing assay, and flow cytometry analysis.

Results: HCC was divided into 2 anoikis subtypes, and the subtypeB had a better prognosis. An anoikis prognostic model based on 12 (COPZ2, ACTG2, IFI27, SPP1, EPO, SLCO4C1, RAB26, STC2, RAC3, NQO1, MYCN, HSPA1B) risk genes is important for survival and prognosis. Significant differences were observed in immune cell infiltration, TME, and drug sensitivity analysis between the risk groups. SLCO4C1 was downregulated in HCC. SLCO4C1 downregulation promoted the proliferation, invasion, migration, and apoptosis of HCC cells. The tumor-suppressive role of SLCO4C1 in HCC has been confirmed.

Conclusions: Our study presents a novel anoikis classification method for HCC that reveals the association between anoikis features and HCC. The anoikis feature is a critical biomarker bridging tumor cell death and tumor immunity. In this study, we provided the first evidence of SLCO4C1 functioning as a tumor suppressor in HCC.

## INTRODUCTION

Among the malignant tumors originating from the liver, hepatocellular carcinoma accounts for the largest proportion. The incidence of hepatocellular carcinoma is related to many factors, including personal lifestyle, genetics and so on. Excessive drinking, hepatitis virus infection and carcinogens in food are the main causes of hepatocellular carcinoma [1]. According to statistics, there are more than 250 million patients with hepatitis virus infection worldwide, of which about 5% of the patients will develop hepatocellular carcinoma [2]. Furthermore, cirrhosis, fatty liver, and diabetes increased the likelihood of HCC [3].

For early hepatocellular carcinoma, the main treatments include surgery and interventional therapy. In cases of advanced liver cancer, radiotherapy and chemotherapy are usually used. Immunotherapy has emerged as a novel therapeutic approach for HCC [4]. Radical resection is the most selected modality in early HCC patients. For inoperable cases, interventional therapy, such as transcatheter radiofrequency ablation and alcohol injection, is also used as a common treatment. Radiotherapy can kill cancer cells by electromagnetic radiation and can effectively treat advanced HCC. Chemotherapy is also an effective treatment, but at present, the effect of chemotherapy on HCC is not good, at the same time, it has some side effects. Immunotherapy is a new treatment in recent years, which can effectively treat HCC by activating the immune system to attack tumor cells [5, 6]. Targeted therapy for immune checkpoints has also made some progress [7]. Despite the availability of numerous treatment options for HCC, the prognosis remains unfavorable, and a considerable number of patients will relapse, which brings more complexity to the treatment of HCC. At present, the popular concept of precision medicine is gradually applied in the treatment of tumor. Mainly comprehensive genomics, transcriptome, proteomics and other aspects of information analysis, strive to grasp the individual differences of patients with diseases, to find a strong basis for treatment [8, 9].

The mechanism of anoikis is that the discontact between the cell and its matrix induces death [10]. It was initially found in mammals that when cells are separated from the interaction of their matrix, it triggers the mechanism of anestrous apoptosis and promotes the elimination of abnormal cells [11]. In normal cells, anoikis plays a role in tumor inhibition because of its mechanism. However, tumor cells can avoid the effects of apoptosis through different mechanisms, and cancer cell proliferation and metastasis will be uninhibited [12]. Apoptosis is an important target in anti-tumor therapy. There are many ways for tumor cells to avoid anoikis. It can promote its own growth by increasing GFR signal pathway and up-regulating hepatocyte growth factor receptor and EGFR. Inhibiting the function of apoptosis-related pathways is another common way for tumor cells to escape from nesting. The Bcl-2 family suppresses the activation of apoptosis-related proteins [13, 14]. At present, the study of anoikis has always been a hot research field in tumor therapy. Anoikis also affects the immunogenicity of tumor cells, which affects their ability to recognize and clear cancer cells.

SLCO4C1 is a carrier protein belonging to the SLCO family. It is a human kidney-specific organic anion transport peptide [15, 16]. SLCO4C1 in head and neck cancer was a tumor suppressor gene [17]. Interference with SLCO4C1 expression caused inactivation of PI3K/Akt pathway causing apoptosis in endometrial cancer [18]. SLCO4C1 has been implicated in tumor progression, there may be heterogeneity in the role in each tumor. Currently, the principle of how SLCO4C1 acts in HCC has not been reported. HCC can be classified into several anoikis subtypes, and the relationship between anoikis and patient prognosis and immune characteristics remains unclear. Our study aims to identify anoikis subtypes and identify new biological targets that can be used for the diagnosis and treatment of HCC.

## MATERIALS AND METHODS

### Data collection and processing

We logged on to TCGA (https://portal.gdc.cancer.gov/repository) to obtain HCC data. Data from 50 normal samples from 371 HCC samples (374 transcripts) were downloaded for analysis. We downloaded 242 GSE14520 HCC samples from the GEO (https://www.ncbi.nlm.nih.gov/geo/). This study downloaded the ICGC-LIRI-JP data on the official website of ICGC (https://dcc.icgc.org/). The data included 232 liver cancer sample transcriptional groups and their clinical information. After screening patients by inclusion and exclusion criteria, a total of 829 HCC samples were used for subsequent analysis. We obtained 338 anoikis-related genes (ARGs) with a correlation score >1.0 from the GeneCards database (https://www.genecards.org/).

### ssGSEA

ssGSEA is an algorithm used to calculate feature scores. The algorithm can evaluate the enrichment of the eigengene sets in a sample. It can reflect the close correlation between the sample and the gene set [19]. We draw the anoikis enrichment spectrum of each HCC sample according to ssGSEA. The GSVA R packet was used to implement the ssGSEA algorithm.

### Consensus clustering analysis of ARGs

Clustering method is widely used in the classification of cancer subtypes. Clustering can classify all contained samples into several subtypes through different sets of datasets, and compare the differences between subtypes [20]. It can be used to discover clinical differences between subtypes and subtypes of new diseases. Consistent clustering confirms the rationality of clustering by constantly re-selecting samples, and determines the value of the final clustering number k. In this study, we used 829 samples from three databases for cluster analysis. The “ConensusClusterPlus” package was used to implement analysis. The number of subtypes was divided according to the optimal k value.

### Analysis of WGCNA

Weighted gene coexpression network analysis (WGCNA) is a processing algorithm for complex patterns. This method calculates the genes with highly synergistic changes in clustered expression patterns, and matches these clustering modules with related traits [21]. The differential genes among clusters were determined by WGCNA algorithm to determine the gene modules which were highly related to the nesting score traits. Pearson test was used to analyze correlation between module eigengenes (MEs) and anoikis. The gene set with the most significant correlation was selected among all the modules with *P* < 0.05.

### Construction of anoikis prognostic model for HCC

To exploit the potential of anoikis features in prediction, we developed a risk model. The anoikis prognosis model refers to the prediction of the probability and risk of patient mortality by evaluating the patient’s clinical data, biological features, and pathological features. The anoikis model was constructed based on the genes obtained from the Cox analysis. The model formula is: Risk Score = β1 × X1 + β2 × X2 + … + βn × Xn. In this formula, Risk Score represents the risk score, β1, β2, …, βn represent the feature weights (gene correlation coefficients), and X1, X2, …, Xn represent the feature variables (gene expression levels), which represent the value of each feature. The nomogram chart is a graphical tool for calculating risk scores. It intuitively displays the relationship between specific patient variables and risk outcomes through images. We constructed a nomogram that can be used for the prognostic evaluation of various clinical indicators. The calibration curve was employed to assess the concordance between the predicted and observed outcomes of the nomogram.

### GSEA

Gene Set Enrichment Analysis (GSEA) can calculate the level of enrichment for each gene set [22]. GSEA analysis was conducted on the key genes within the risk model. The analysis of the major enriched pathways between two risk groups was performed using R packages, including “org.Hs.eg.db”, “clusterProfiler”, and “enrichplot”.

### Analysis of immune infiltration

To mine the connection of anoikis features and immune cells, we employed 7 algorithms (TIMER, CIBERSORT, CIBERSORT-ABS, XCELL, MCP-counter, EPIC, QUANTISEQ) [23]. The Pearson’s coefficient was used to assess the association degree between the immune cells. For the above analysis, the R packages “reshape2”, “tidyverse”, “ggplot2”, “ggpubr”, and “ggExtra” were employed. The content of immune checkpoints is closely correlated with tumor survival. For example, tumor patients with high levels of PD-L1 expression usually respond more effectively to immune checkpoint inhibition therapy and have longer survival periods. This study selected several common immune checkpoint genes (ATIC, CTLA4, PDCD1, LAG3, PDCD1LG2, CD47, CD40, CD80, CD86) for analysis of their correlation with the anoikis risk score. Correlation analysis was performed by “limma” and “corrplot” R packages.

### Identify TMB differences between risk groups

Tumor mutation burden (TMB) is a reflection of the genomic diversity of tumor cells.

Tumor cells develop mutations more frequently, which could be attributed to their biological characteristics such as higher DNA replication error rates, or due to their exposure to environmental factors such as carcinogens or viruses [24]. A high TMB often indicates a higher frequency of gene mutations, which could result in faster tumor growth, increased malignant behavior, and poor treatment outcomes. Conversely, a low TMB suggests fewer gene mutations, which could lead to a slower tumor growth rate, lower degree of malignancy, and more promising therapeutic effects.

The TCGA-LIHC cohort was used to analyze the relationship between TMB and risk scores. The “maftools” software package was used to identify genes with higher mutation frequency in the risk groups. Additionally, we used the “limma” and “ggpubr” software packages to complete the differential analysis.

### Difference analysis of TME

The environment in which tumor cells survive is known as the tumor microenvironment (TME). Various components in the TME can interact with each other and affect tumor function. The cellular composition of the tumor microenvironment (TME) primarily consists of tumor, immune, and stromal cells [25].

The extracellular matrix (ECM) is also essential in tumor progression. Tumor cells can change the composition and structure of the ECM to adapt to the growth needs of the tumor. ESTIMATE is a method for calculating the proportion of different cell populations in the TME, which can be used to estimate the composition of the TME. ESTIMATE can be used to calculate the immune cells and stromal cells of the sample and the ESTIMATE score. The scoring reflects the infiltration abundance of the corresponding cells. The ESTIMATE algorithm can be implemented through the R “estimate” package.

### Drug sensitivity analysis

The prognosis of cancer patients is similarly affected by drug sensitivity. By evaluating the sensitivity of tumors to certain drugs, drug sensitivity analysis can guide doctors to develop more personalized and effective treatment plans, so as to improve treatment results and patient prognosis. Drug sensitivity analysis can help determine which patients are suitable for which drugs, and how to use these drugs at different stages. We downloaded drug information from the GDSC website and used “oncoPredict” and “parallel” in the R software to predict drug sensitivity in patients in the risk groups. The *p*-value filtering condition was set to *P* < 0.001.

### Patient sample collection

Fresh tumors and corresponding adjacent tissues from 30 HCC patients were collected in this study. All the patients signed a written informed consent form. The Medical Ethics Committee of Anhui No.2 Provincial People’s Hospital approved this study.

### RT-qPCR

Total tissue RNA extraction was performed according to the RNA Purification Kit manual. The cDNA was synthesized using the EvoM-MLVRTPremixcDNAS synthesis kit (Accurate Biotechnology, China). SYBR Green Premix Pro Taq HS qPCR Kit (Accurate Biotechnology, China) was used in real-time quantitative polymerase chain reaction process. The primer sequences used are as follows: GAPDH: F, 5-CCACTCCTCCACCTTTG-3, R, 5-CACCACCCTG TTGCTGT-3 SLCO4C1: F, 5-TTCCCTGACTGG CCTGATTTC-3, R, 5-GCAAGCCATCTCGG CTTATG-3.

### Cell culture and transfection

The Cell Bank of the Chinese Academy of Sciences provides human HCC cell lines Hep3B, HepG2, MHCC-LM3, SMMC-7721, MHCC97-H, PLC/PRF/5 for the research. Cells were cultured using DMEM (Gibco, USA) supplemented with 10% fetal bovine serum. The required environment for a conventional cell culture incubator is 5% CO2 at 37°C. Purchased short hairpin RNA (shRNA, Zebra Biologics, China) was transfected into HCC-LM3 and HepG2 according to the rules of instructions. The ShRNA sequence is as follows: SLCO4C1-sh1: 5-GCCATAAGTGTTAC TTGTAAA-3, SLCO4C1-sh2: 5-GCTACGATATTT CATTCTGTT-3.

### Western blotting

Total protein was extracted using 1% RIPA lysates (Servebio, Wuhan, China). The protein was transferred from the SDS-PAGE gel to NC membrane. Subsequently, the primary antibodies (SLCO4C1, 24584-1-AP, Proteintech, China; β-actin, 81115-1-RR, Proteintech, China) were incubated with the NC membrane overnight at 4°C. After incubation, chemiluminescence kit (ECL) was used to develop chromogenic protein.

### Cell proliferation assay

Transfected HCC cells were plated in 96-well plates at a density of 4 × 10^3^ cells per well, and 100 μl of complete culture medium was added to each well. Four holes were repeatedly added to each sample. 10 ul CCK-8 (Biomark, Shanghai, China) was added after 24 h, 48 h, 72 h and 96 h in 37°C incubator. After incubated in cell incubator for 2 hours, the absorbance of OD450 was detected by enzyme labeling instrument.

After successful transfection, HCC cells were seeded in 6-well plates at a density of 1 × 10^3^ cells per well and maintained in culture for 12 days. After washing with PBS, the samples were fixed in 4% paraformaldehyde for 15 minutes and subsequently stained with 0.1% crystal violet for 10 minutes. After fully rinsing, the cell colonies were counted under the microscope and pictures were taken.

### Analysis of cell invasion and migration

After a 24-hour transfection period, the cells were collected and then seeded onto the upper compartment of a 24-well plate that had been pre-coated with an 8 μm matrix gel (Corning, USA). In the lower part, 600 μl DMEM complete medium of 20%FBS was added. After a 24-hour incubation at 37°C, the cells were fixed with 4% paraformaldehyde for 20 minutes, followed by staining with 0.1% crystal violet for 10 minutes, and counted under the microscope after full washing.

The transfected cells were plated in a 6-well (4 × 10^5^ cells/well). Subsequently, a scratch was created in each well using a sterile 10 ul pipette tip. The superfluous cells in the upper layer were washed by PBS and cultured with DMEM containing serum. Cells were washed with PBS and then cultured in serum-containing DMEM. After 48 hours, a microscope was used to take pictures and calculate the change of the area of scratches.

### Apoptosis assay

The Annexin V-APC/7-AAD kit (KeyGEN BioTECH, China) was used for apoptosis detection in the cells. First, 5 μl 7-AAD dye solution was added to 50 μl Binding Buffer and mix well. The cells were collected and mixed with the above-mentioned 7-AAD staining solution, and the 5~15 min was protected from light at room temperature. After the reaction, a mixture of 450 μl Binding Buffer and 5 μl Annexin V-APC was added. Subsequently, the mixture was incubated at room temperature for 15 minutes. Cellular analysis was performed using cytoFLEX flow cytometry equipment (Beckman Coulter, USA).

### Statistical analyses

R (version 4.2.2), GraphPad Prism8, and SPSS25 were utilized in this study. Independent *t*-tests were performed to assess the differences between groups. Wilcoxon test was used to analyze the immune therapy response between groups. The significance level was defined as *P* < 0.05.

### Data availability statement

Publicly available datasets were used in this study. These data can be found here: TCGA database (http://portal.gdc.cancer.gov/repository), ICGC database (https://dcc.icgc.org/), GEO database (GSE14520) (http://www.ncbi.nlm.nih.gov/geo/), GeneCards (https://www.genecards.org/), IMvigor210 cohort (http://research-pub.gene.com/IMvigor210CoreBiologies/), CIBERSORT (https://cibersortx.stanford.edu/about.php), TCIA database (https://tcia.at/), GDSC database (https://www.cancerrxgene.org/), TIDE database (http://tide.dfci.harvard.edu). The data that support the findings of this study are available from the corresponding author upon reasonable request.

## RESULTS

### Identification and clinical significance of classification related to anoikis in HCC

**Figure 1:**
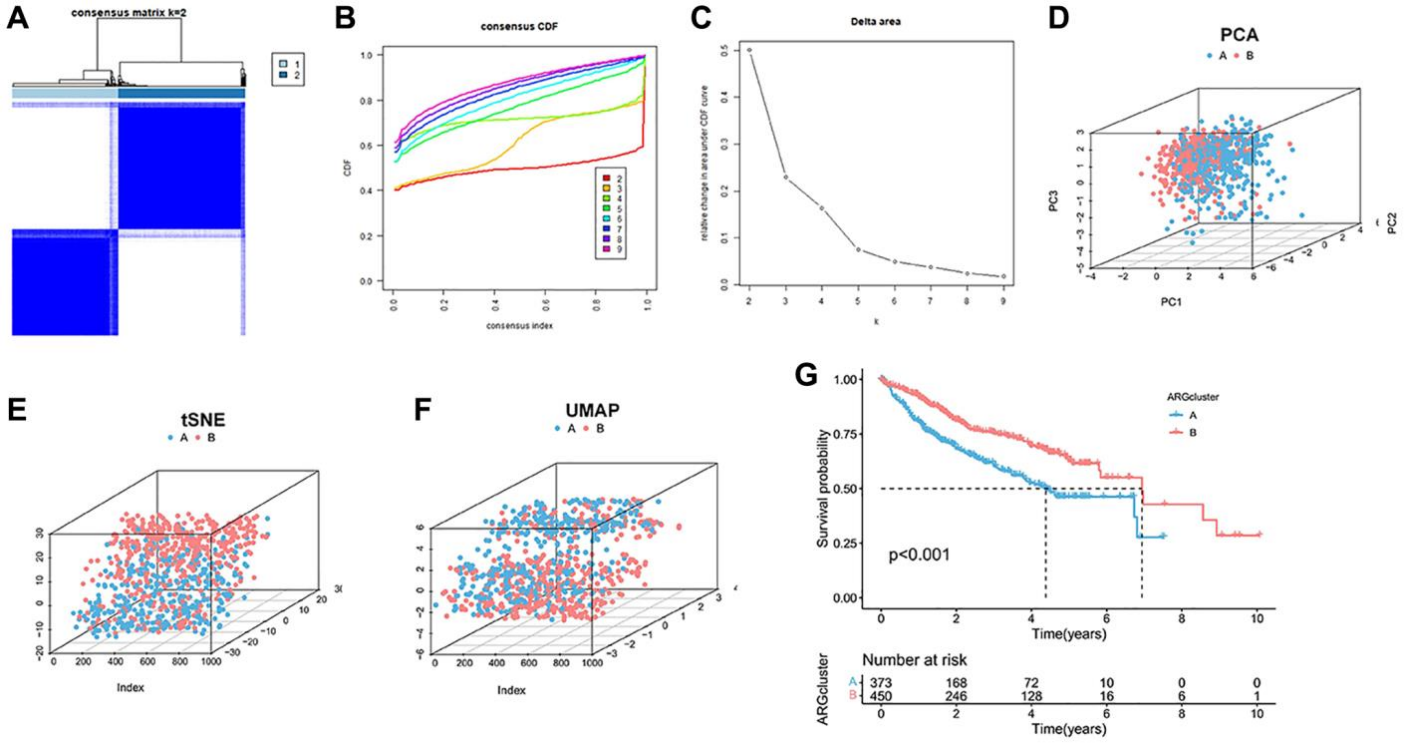
**Cluster analysis of anoikis in HCC cohort.** (**A**) The HCC data sets in the three queues are divided into two different clusters. (**B**) Consensus clustering cumulative distribution function (CDF) for k = 2~9. (**C**) The relative change of the area under the CDF curve from k = 2 to 9. (**D**–**F**) PCA, tSNE and UAMP diagrams show that the cluster can distinguish patients according to the expression profile of HCC dataset. (**G**) Kaplan-Meier curve survival analysis among different clusters.

The expression of anoikis was extracted from 338 downloaded samples of HCC patients integrated with TCGA, GSE14520 and ICGC. A total of 283 homing gene expression files were obtained from 828 samples. Cluster analysis was performed to classify the samples into two distinct subtypes, including 376 clusterA and 452 clusterB (Figure 1A–1C). Subsequently, according to the dimension reduction analysis of typing, the anoikis-related genes could well distinguish the two cluster (Figure 1D). The results of tSNE and UMAP also confirmed that there were some differences in the distribution of the two cluster types (Figure 1E, 1F). Furthermore, patients classified as clusterB showed a poorer prognosis compared to those classified as clusterA (Figure 1G). ClusterA was much more anoikis-resistant than it was in clusterB.

### TME features and immune infiltration between the anoikis subgroups

**Figure 2:**
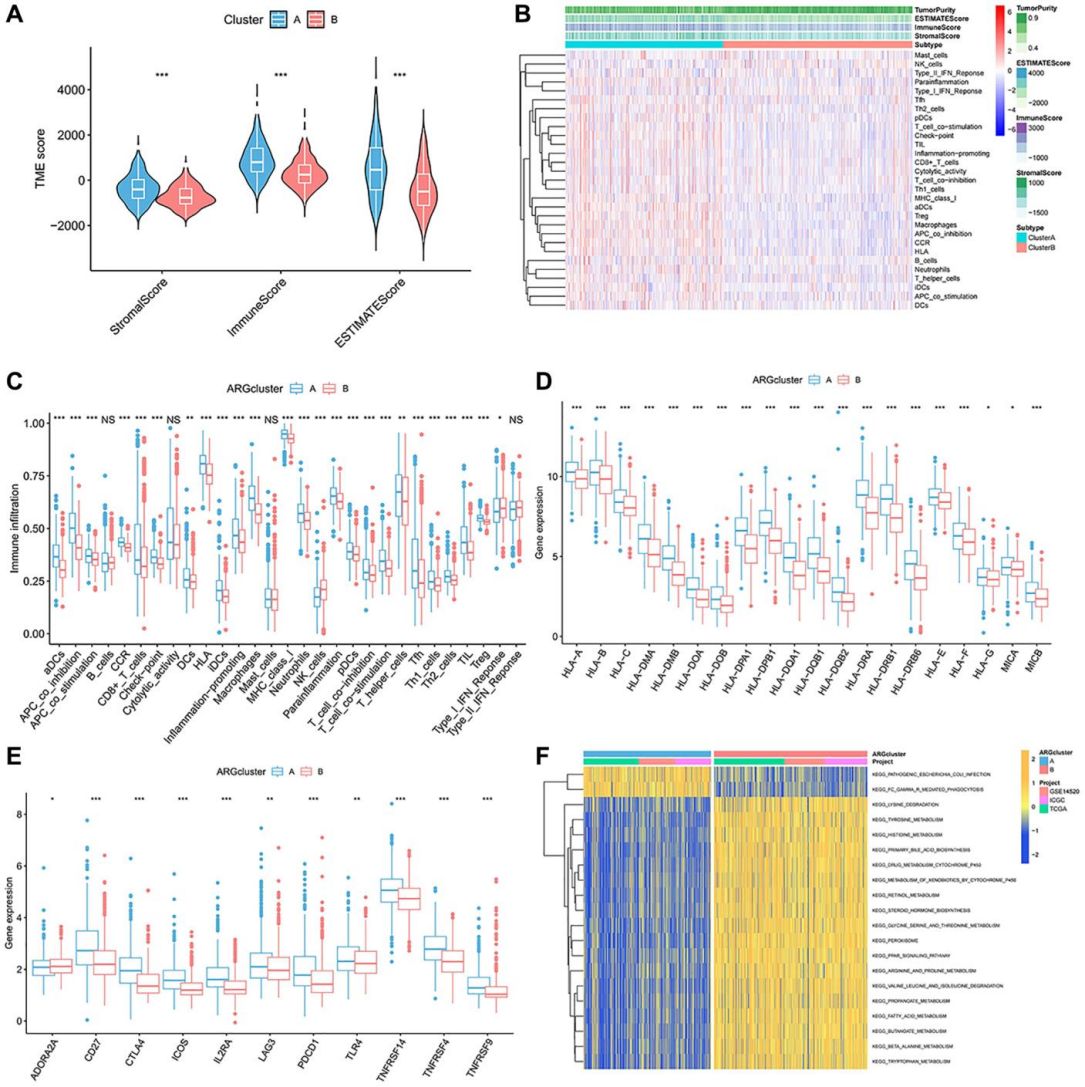
**Immune-infiltration landscape in the two anoikis subtypes.** (**A**) The TME score in the A anoikis subtype. (**B**, **C**) The native distribution of 29 common immune cells in the anoikis subpopulation. (**D**) Expression content of the DMHC family genes in each subset. (**E**) Analysis of the abundance of immunosuppressive molecules in subsets. (**F**) GSVA analysis of the pathways enriched in the subclusters. ^*^p < 0.05, ^**^p < 0.01, ^***^p < 0.001.

To explore the immune infiltration and TME distinction in the anoikis subtype population, we calculated the corresponding feature scores. In terms of scoring, the immune, stromal, and ESTIMATED scores were lower in subgroupB than in subgroupA (Figure 2A). SubgroupB also contained less immune cells than subgroupA (Figure 2B). Quantitative analysis of 29 immune cells showed high levels of APC costimulatory cells, CD8+ T cells, HLA, iDCs, macrophages, pDCs, T cells co-stimulation, Th cells, and Treg cells in the subgroupA population (Figure 2C). The expression of the HLA family in subgroupA also showed consistency (Figure 2D). We noted that both costimulatory and corepressed cells were highly enriched in the anoikis resistant subpopulation. Similarly, the corepressor was more enriched in subgroupA (Figure 2E). This suggests a combined role of immunosuppression and stimulation in the anoikis resistance subgroup. GSVA analysis found that subgroupA was associated with the phagocytosis pathway, while subgroupB was involved in multiple amino acid metabolism and biosynthesis (Figure 2F).

### Construction and verification of aniokis prognostic model

**Figure 3:**
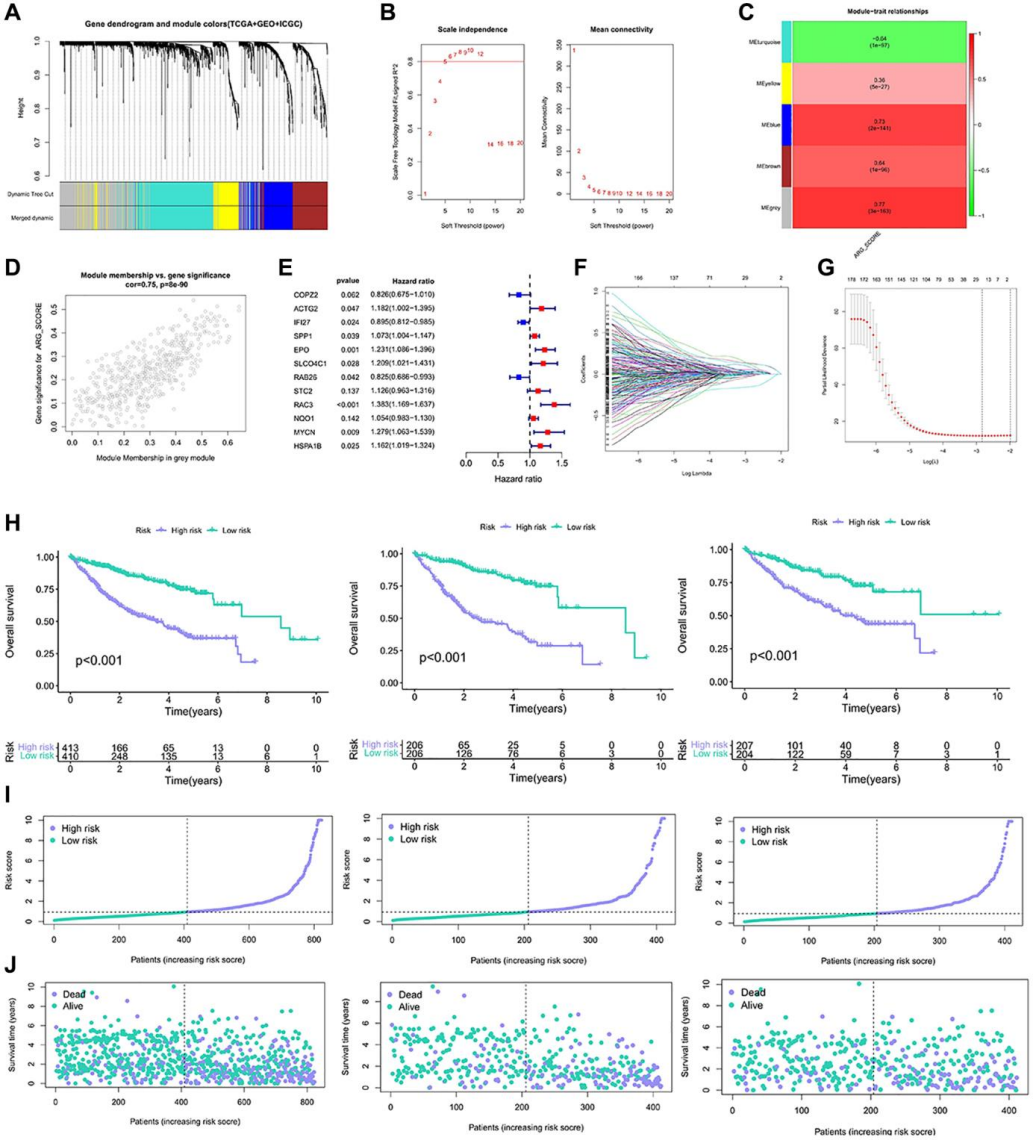
**Construction of the model based on the anoikis classification.** (**A**, **B**) Co-expression network established by TCGA-LIHC database, GSE14520 data set and ICGC data. (**C**) Heatmap of the correlation between eigenes and anoikis scores for grey modules. (**D**) Grey module has the strongest correlation with anoikis (Cor = 0.75, *P* = 8e−90). (**E**) The 12 gene expression characteristics based on the type of anoikis were selected using the LASSO-Cox model. (**F**, **G**) Optimize the cross validation of parameter selection in LASSO model. (**H**) Kaplan-Meier curve survival analysis of HCC patients in all samples, train group and test group. (**I**) Risk curves of all samples, train groups and test groups. (**J**) Risk status diagram of all samples, train group and test group.

To identify differential genes associated with anoikis, we identified differential genes between the two genotypes. 6830 differentially expressed genes (EDGs) were identified. Then, these DEGs are classified as distinct modules passing through WGCNA (Figure 3A, 3B). The anoikis score was calculated using the ssGSEA. Among all the modules examined, the grey module showed a strong correlation with the anoikis score (R^2^ = 0.77, *P* = 33e−163) (Figure 3C, 3D). We developed a risk model based on anoikis clustering to predict prognosis. Firstly, 192 genes were obtained by univariate Cox analysis of 491 genes (Supplementary Table 1). After univariate analysis, the genes were analyzed by Lasso-Cox, and 12 gene expression characteristics based on aniokis were selected (Figure 3E, 3F). The 12 genes information were shown in Table 1. The survival curve demonstrated that the high-risk group exhibited a poorer overall survival (OS) (Figure 3G). The same results are also shown in the train group and the test group (Figure 3H). The elevated score corresponds to an increased proportion of high-risk patients and an increased risk of death (Figure 3I). The correlation was found between the risk survival status analysis and increased mortality, and the OS was relatively short (Figure 3J). The anoikis model showed a potential prognostic value.

**Table 1:** 12 Gene information of anoikis risk models.

**Id**	**Coef**	**HR**	**HR.95L**	**HR.95H**	***p*-value**
COPZ2	−0.191526	0.8256981	0.6751926	1.0097525	0.0621203
ACTG2	0.1675359	1.1823877	1.0020956	1.3951171	0.0471708
IFI27	−0.1111601	0.8947955	0.8124654	0.9854684	0.0239951
SPP1	0.0703641	1.0728987	1.0036794	1.1468918	0.0386497
EPO	0.2079613	1.2311655	1.0860713	1.3956437	0.001152
SLCO4C1	0.1896167	1.2087862	1.0208163	1.4313683	0.0278864
RAB26	−0.1921739	0.8251634	0.6858819	0.9927285	0.0416165
STC2	0.1185894	1.1259075	0.9631719	1.3161385	0.1365227
RAC3	0.3244314	1.3832439	1.1685828	1.6373369	0.0001629
NQO1	0.0523483	1.0537427	0.9825589	1.1300835	0.1423996
MYCN	0.2460688	1.2789875	1.0631165	1.5386922	0.0090842
HSPA1B	0.1497878	1.1615877	1.01944	1.3235561	0.0245091

### Functional analysis and anoikis model independent predictive value analysis

**Figure 4:**
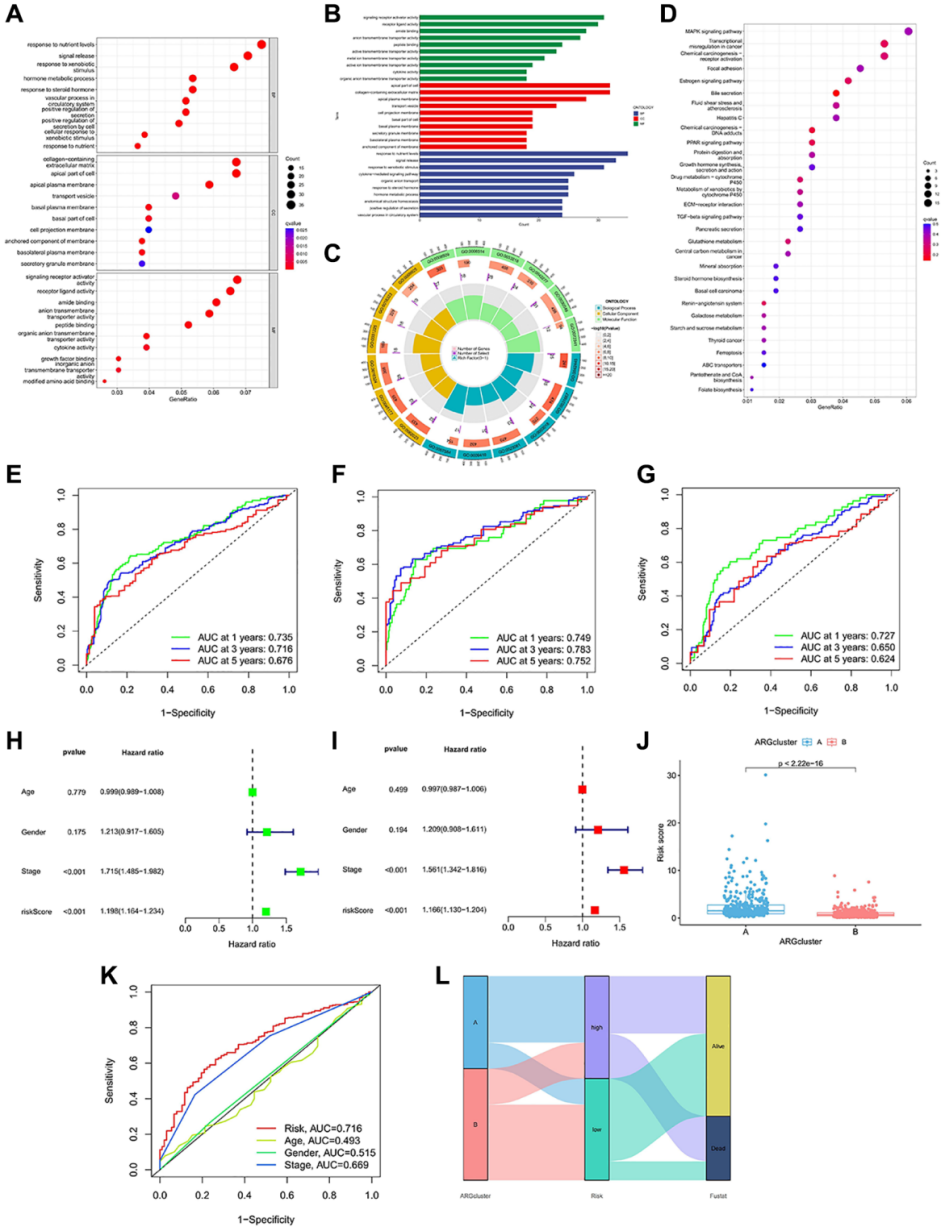
**Enrichment analysis and prognostic model construction based on anoikis DEGs.** (**A**–**C**) GO pathways enriched between the high-risk and low-risk groups. (**D**) KEGG pathways enriched between high-risk and low-risk groups. (**E**–**G**) ROC curves of all samples, train group and test group. (**H**, **I**) Univariate and multivariate Cox regression analysis showed that the risk score based on anoikis correlation cluster was an independent prognostic factor affecting the prognosis of HCC patients. (**J**) There are significant differences between cluster A and cluster B in risk score. (**K**) The ROC prediction curve of the anoikis model. (**L**) Sankey diagram reveals the potential relationship between clustering, risk score and survival status.

To further analyze the gene functions of prognostic DEGs, we conducted enrichment analysis. GO analysis revealed that risk genes were primarily enriched in biological processes (BP) related to nutrient responses. It is enriched in kinesin complex and other pathways in the process of cell (Cellular Component, CC). In the process of molecular function (MF), it is mainly enriched in the pathway activated by receptors (Figure 4A–4C). KEGG indicated that ARGs were prominently enriched in signaling pathways such as “MAPK signaling pathway,” “transcriptional dysregulation in cancer,” “chemical carcinogenesis-receptor activation,” “focal adhesion,” “chemical carcinogenesis-DNA adducts,” “PPAR signaling,” and others (Figure 4D). High-risk subtypes showed significant enrichment in pathways such as “cell cycle”, “DNA replication”, “ECM receptor interaction” and “glycosaminoglycan biosynthesis” (Supplementary Figure 1A). The main enrichment pathways in low-risk subtypes were “replenishment and condensation cascade”, “drug metabolism cytochrome P450”, “cytochrome P45”, “retinol metabolism” and “steroid hormone biosynthesis” (Supplementary Figure 1B).

Moreover, the accuracy of the anoikis model was also validated. In the complete dataset of HCC, the AUC values for 1-, 3- and 5-year OS were 0.735, 0.716, and 0.676 (Figure 4E). In the training group, the AUC values for 1-, 3- and 5-year OS were consistent with the complete dataset, measuring at 0.749, 0.783, and 0.752 (Figure 4F). Similarly, the test set exhibited consistent results, with AUC values of 0.727, 0.650, and 0.624 for 1-year, 3-year, and 5-year OS (Figure 4G). The anoikis model we constructed has shown differences in survival, and further independent prognostic analysis was carried out. Stage and risk score have been identified as prognostic factors in this study (Figure 4H, 4I). When comparing the risk differences between the two anoikis types, we observed a significantly lower risk in clusterB compared to clusterA (Figure 4J). The predictive value of the risk score and stage was also reflected in the ROC curve (Figure 4K). The Sankey chart illustrates that the majority of patients in the survival status belong to the low-risk group, while they consisted mainly of clusterB patients (Figure 4L). The majority of deceased patients exhibited higher risk scores and were associated with clusterA.

### The immune landscape of the anoikis risk groups

**Figure 5:**
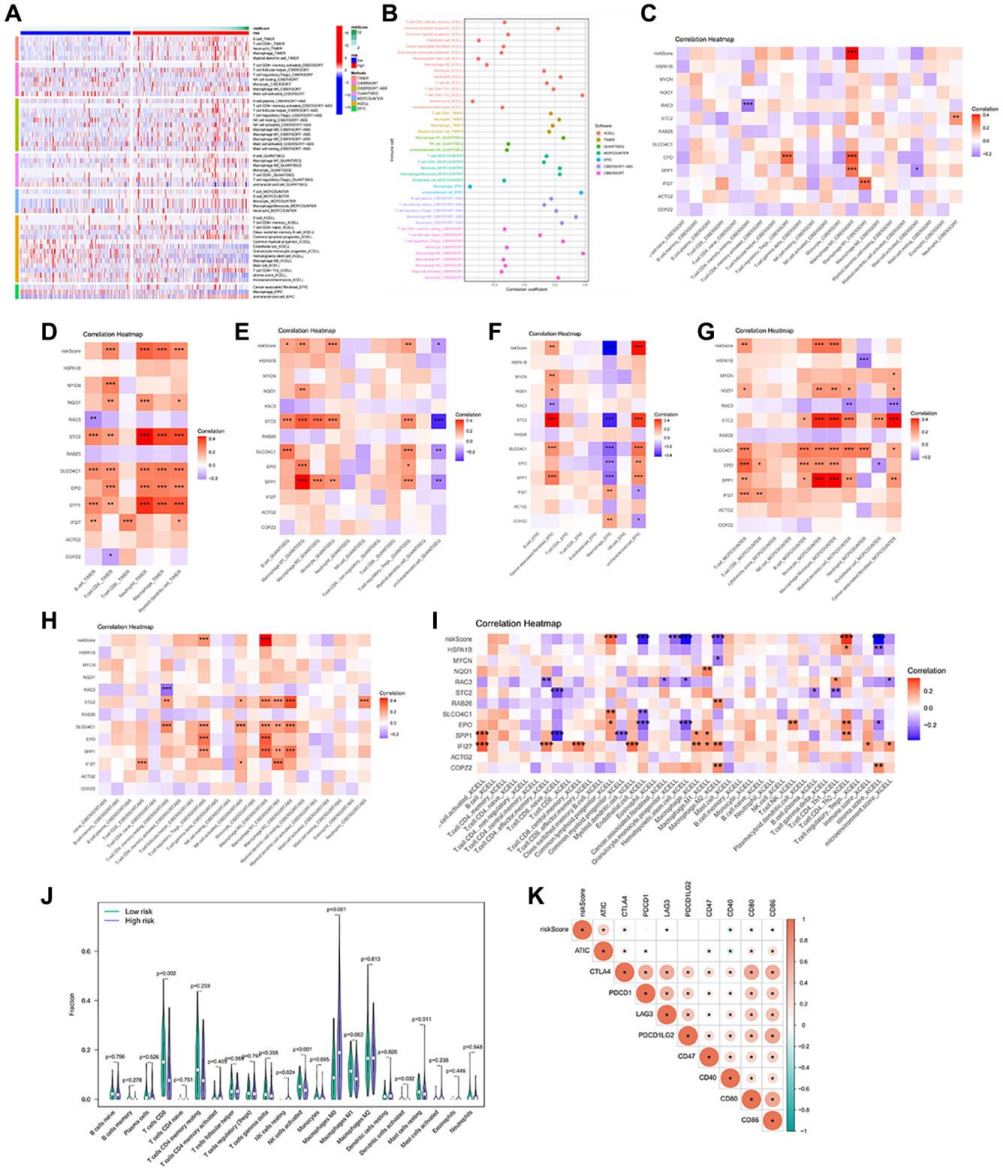
**Immune infiltration profile in the risk groups.** (**A**, **B**) 7 algorithms evaluated the immune cell infiltration between the HCC risk groups. CIBERSORT (**C**), TIMER (**D**), QUANTISEQ (**E**), EPIC (**F**), MCP-counter (**G**), CIBERSORT-ABS (**H**), XCELL (**I**) to calculate the correlation of signature genes and risk scores with each immune cell. (**J**) The abundance of immune cells between the high and low risk groups. (**K**) Heatmap of correlation between Immune checkpoint genes and risk score. ^*^p < 0.05, ^**^p < 0.01, ^***^p < 0.001.

To better understand the potential mechanism of action of the anoikis, we investigated the correlation between anoikis and immune cell infiltration. The immune cell thermogram showed that the immune infiltration was more significant in the high-risk group (Figure 5A, 5B). We examined the correlation of signature genes and risk scores with immune cells, and most of the results were significant (Figure 5C–5I). The infiltration of CD8T cells, activated NK cells and resting mast cells was higher in the low risk group (Figure 5J). Immune checkpoint is an important target for immunotherapy. The analysis revealed a positive correlation between ATIC, CTLA4, LAG3, CD80, CD86, and the risk score (Figure 5K). These results suggest that the anoikis risk model is closely related to immune infiltration and may affect the responsiveness of immunotherapy.

### Analysis of the immunotherapy responsiveness

**Figure 6:**
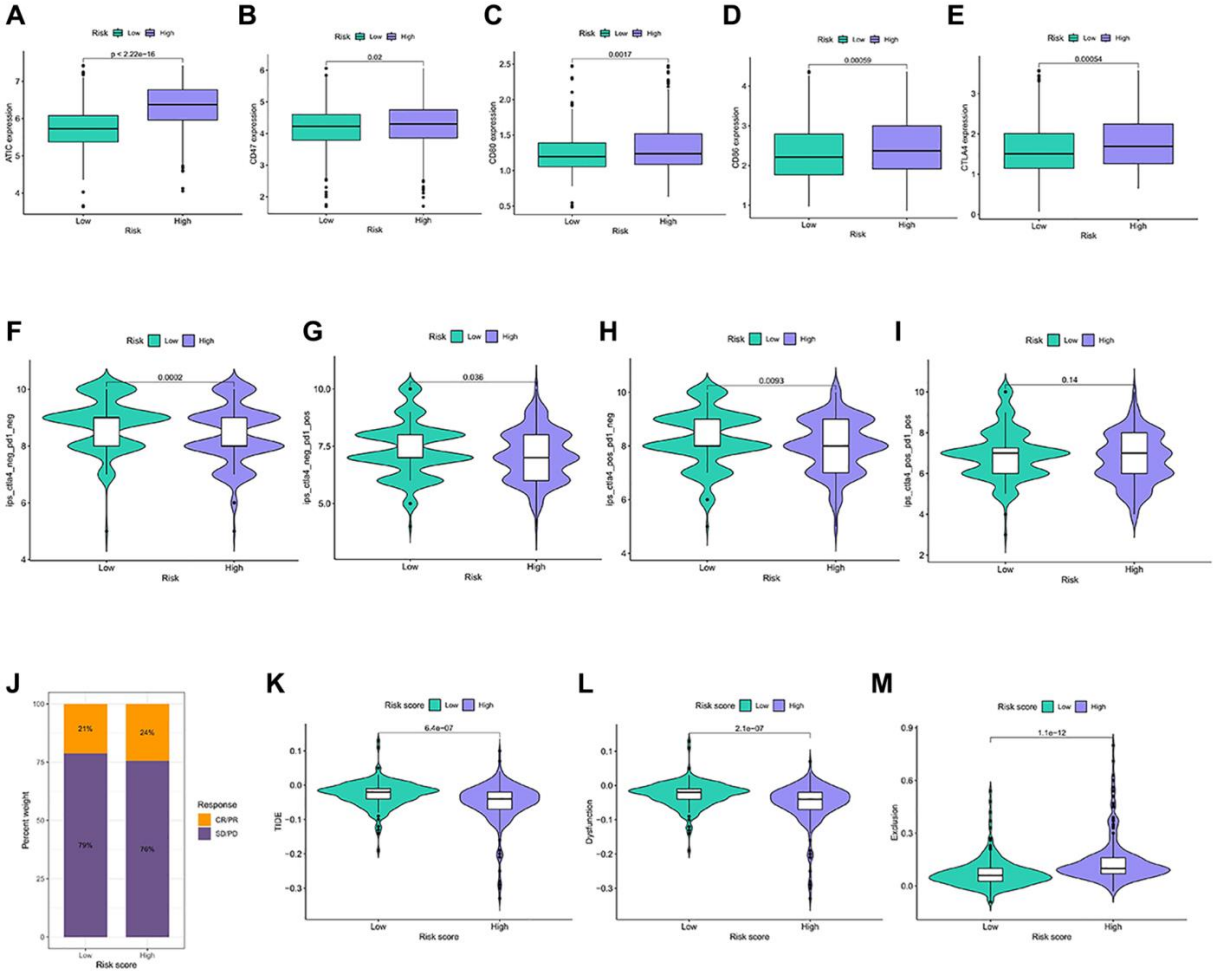
**Immunotherapy response in different risk groups.** (**A**–**E**) Expression of the 5 immune checkpoint genes in the risk groups. (**F**–**I**) IPS in the four immunotherapy groups in the risk groups. (**J**) Statistics of the treatment degree of patients in IMvigor210 cohort. (**K**–**M**) TIDE scores were calculated for immunotherapy responsiveness in the risk groups.

Immune checkpoints are universally selected targets for the development of immunotherapeutic agents, and understanding the expression of their related genes helps in predicting the efficacy of immunotherapy. ATIC, CD47, CD80, CD86, and CTLA 4 were more abundant in the high-risk group (Figure 6A–6E). Patients in the higher-risk group achieved a lower IPS score (Figure 6F–6I). In the independent immunotherapy outcome IMvigor210 cohort, the proportion of patients achieving CR/PR was relatively high in the high-risk group (Figure 6J). The TIDE score reflects the clinical response power of immunotherapy, and obtains higher TIDE and Dysfunction score and lower Exclusion score, which may indicate a poor treatment effect. The presence of relatively low TIDE and Dysfunction scores was observed in the high-risk group, compared with higher Exclusion scores (Figure 6K–6M). Patients in the high-risk group can get a better treatment effect.

### Analysis of TMB, TME and drug sensitivity in risk groups

**Figure 7:**
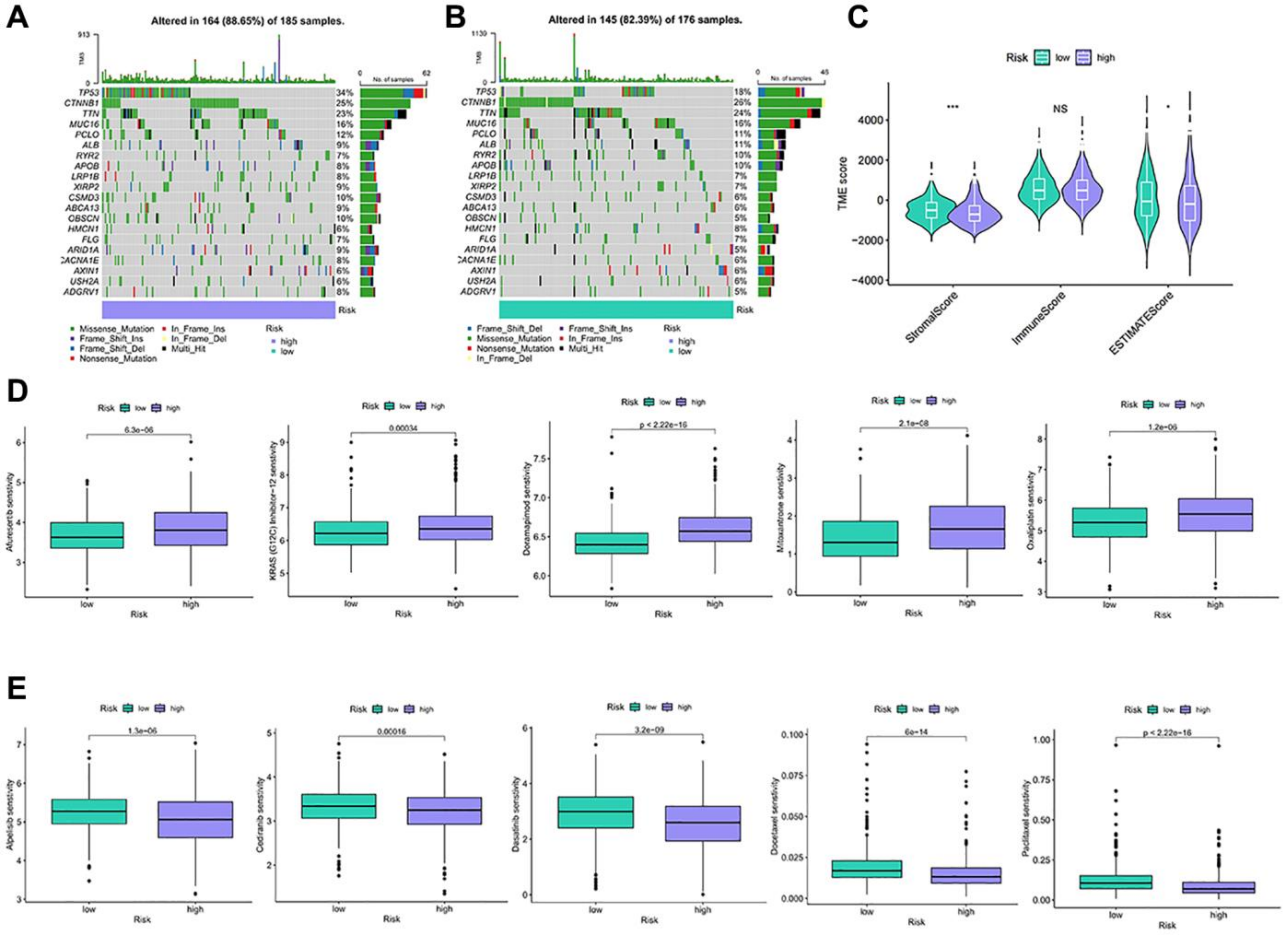
**TMB and drug sensitivity between different risk characteristic groups.** (**A**, **B**) The diagram shows gene mutations in high and low risk groups. (**C**) Analysis of tumor microenvironment components between risk characteristic groups. (**D**, **E**) Sensitivity analysis of chemotherapeutic drugs between different risk groups. NS *p* > 0.05, ^*^p < 0.05, ^**^p < 0.01, ^***^p < 0.001.

We analyze the relationship between the anoikis risk model and TMB. TP53, CTNNB1, TTN, MUC16, PCLO were identified as being more frequently observed in the high-risk group (Figure 7A). The main type of mutation is missense mutation. The same pattern occurred in the low-risk group (Figure 7B). High-risk stromal scores and ESTIMATE scores were low, while the immune scores remained relatively stable between the two groups (Figure 7C). Low ESTIMATE score may be related to prognosis and therapeutic effect. Afuresertib, KRAS(G12C) Inhibitor-12, Doramapimod, Mitoxantrone, and Oxaliplatin showed higher sensitivity in high-risk patients (Figure 7D). However, patients in the high-risk exhibited lower sensitivity to Alpelisib, Cediranib, Dasatinib, Docetaxel, and Paclitaxel (Figure 7E). Thus, low-risk patients will benefit from these drugs.

### Construct a nomogram to predict HCC survival and identify biological markers

**Figure 8:**
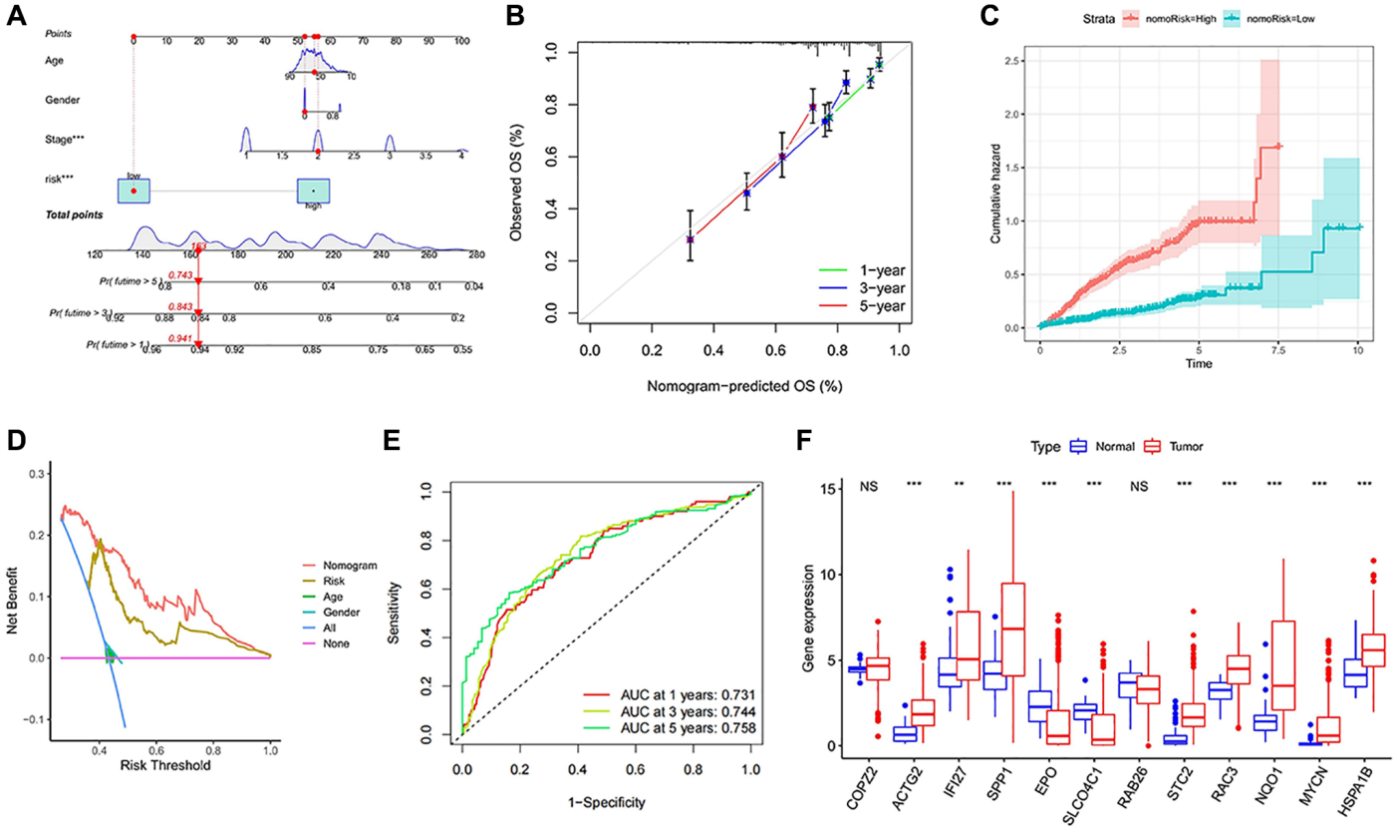
**Establishment and evaluation of survival prediction nomogram.** (**A**) The 1-, 3- and 5-year survival rates were predicted by combining the risk score and other clinicopathological parameters. (**B**) The calibration curve shows the 1-, 3- and 5-year overall survival rate prediction of the nomogram we established. (**C**) The accumulated risk shows differences in the construction of the nomogram. (**D**) Decision curve analysis of 5-year overall survival rate. (**E**) The ROC curve of the nomogram. (**F**) Expression levels of the 12 signature genes in the TCGA data. NS *p* > 0.05, ^*^p < 0.05, ^**^p < 0.01, ^***^p < 0.001.

To provide a more intuitive prediction of patient prognosis and survival time, a nomogram was developed (Figure 8A). The high-risk group exhibited a significantly higher cumulative hazard (Figure 8B). The predicted results from the nomogram exhibit a strong similarity to the actual probabilities (Figure 8C). DCA demonstrates that the nomogram and risk value provide greater net benefits (Figure 8D). The ROC curve of Nomogram suggested a higher reliability (Figure 8E). All of these indications suggest that the risk model and nomogram have high value in terms of prognosis. Among all the 12 signature genes, EPO and SLCO4C1 were lower expressed in the HCC data for TCGA (Figure 8F). EPO has been well studied and the biology of SLCO4C1 in HCC has not been elucidated. We selected SLCO4C1 for subsequent studies.

### Downregulation of SLCO4C1 expression in HCC

**Figure 9:**
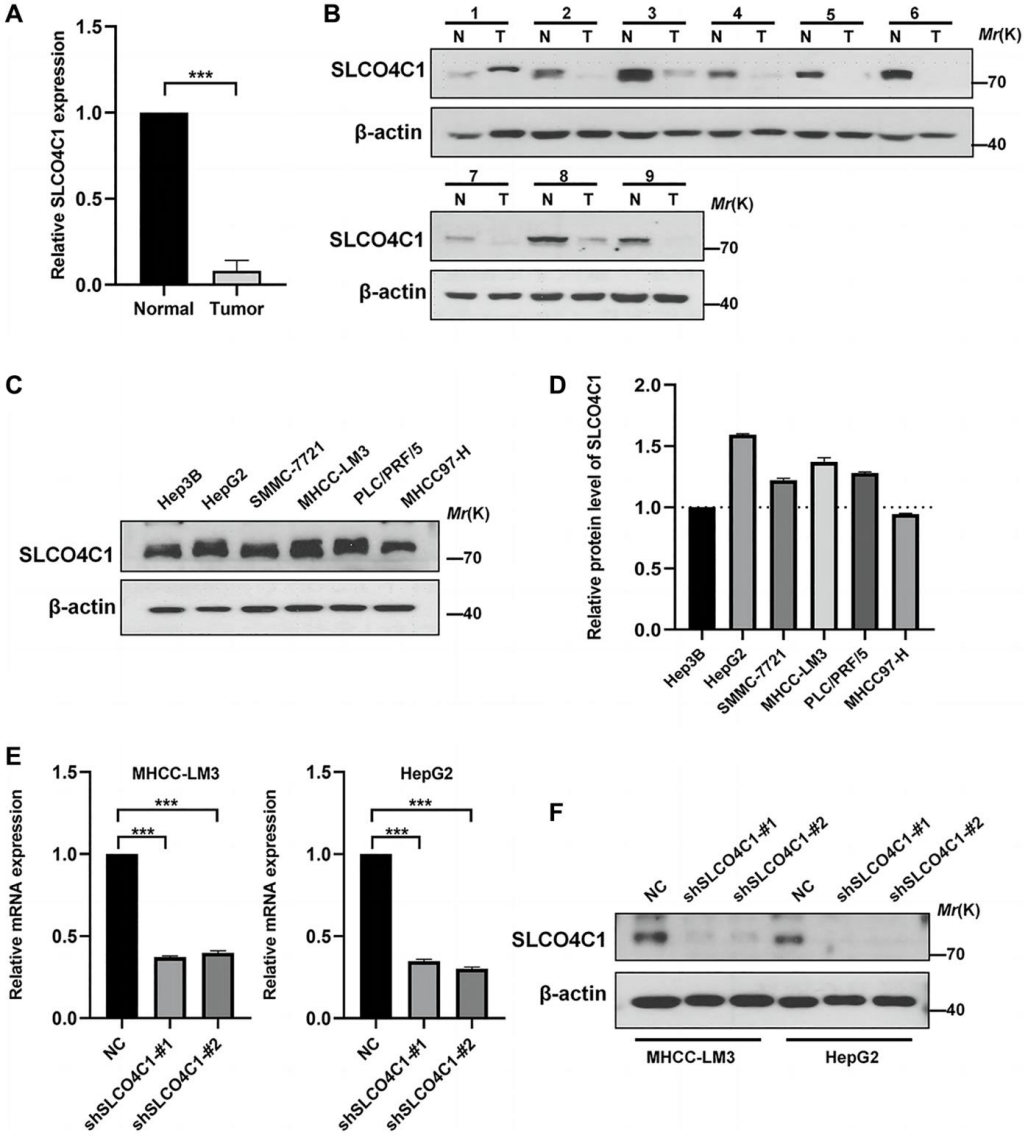
**Expression of SLCO4C1 in HCC tissues and cell lines.** (**A**) 30 SLCO4C1 mRNA expression levels in paired HCC tissues versus normal tissues. (**B**) 9 protein expression levels of SLCO4C1 in paired HCC tissues versus normal tissues. (**C**, **D**) The protein expression levels of SLCO4C1 in 6 common HCC cell lines. (**E**) The mRNA expression levels of SLCO4C1 in the HepG2 and MHCC-LM3 cell lines. (**F**) Protein expression levels of SLCO4C1 in HepG2 and MHCC-LM3 cells transfected with shRNA. ^*^P < 0.05, ^**^P < 0.01, ^***^P < 0.001.

The expression of SLCO4C1 in HCC and normal tissues was assessed using RT-qPCR and Western Blotting. Low mRNA levels of SLCO4C1 in HCC tissues (Figure 9A). The SLCO4C1 protein expression was also downregulated in HCC tissues (Figure 9B). SLCO4C1 expression levels were found to be higher in HepG2 and MHCC-LM3 among the six different HCC cell lines (Figure 9C, 9D). HepG2 and MHCC-LM3 cell lines were selected to transfect SLCO4C1 knockdown specific shRNA for further study. The figure shows that sh1 and sh2 have better inhibitory effect on SLCO4C1 protein expression than NC group in two selected HCC cell lines. The inhibitory effects of sh1 and sh2 on SLCO4C1mRNA level and protein expression level were significant in these two cell lines (Figure 9E, 9F).

### Downregulation of SLCO4C1 promoted the proliferation and invasion and migration of HCC cells

**Figure 10:**
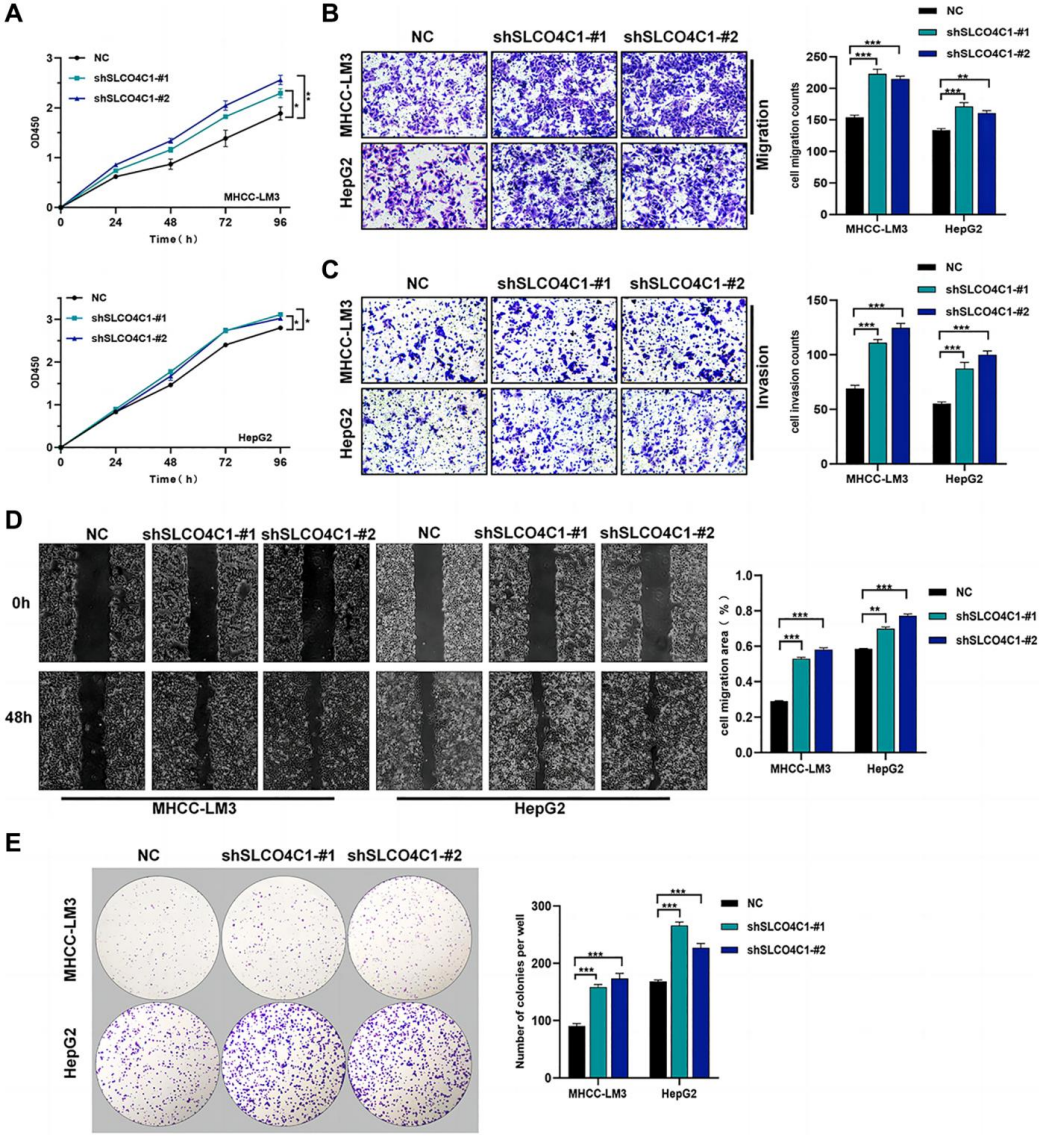
**Effect of sh-SLCO4C1 on proliferation, invasion, and migration of HCC cells.** (**A**) Comparison of the cell proliferation capacity of HepG2 and MHCC-LM3 after SLCO4C1 knockdown. (**B**) Comparison of the invasive capacity of HepG2 and MHCC-LM3 after SLCO4C1 knockdown. (**C**) Comparison of the migration ability of HepG2 and MHCC-LM3 after SLCO4C1 knockdown. (**D**) Scratch healing assay comparing HepG2 and MHCC-LM3 after SLCO4C1 knockdown. (**E**) The proliferation changes of HepG2 and MHCC-LM3 cells after SLCO4C1 knockdown were observed in the colony formation assay. ^*^P < 0.05, ^**^P < 0.01, ^***^P < 0.001.

CCK-8 assays showed significantly higher cell viability in the SLCO4C1-sh1 and SLCO4C1-sh2 than in the normal group (Figure 10A). The invasion assay showed that HCC cells with knockdown SLCO4C1 exhibited enhanced invasion (Figure 10C). The SLCO4C1 knockdown group had a faster scratch healing rate and a stronger migration ability (Figure 10B, 10D). More colony formation was observed in the SLCO4C1-sh1 and SLCO4C1-sh2 groups, and the knockdown of SLCO4C1 increased the proliferative viability of HepG2 and MHCC-LM3 cells (Figure 10E). Reducing SLCO4C1 expression promoted the proliferative viability, invasion, and migration ability of HepG2 and MHCC-LM3 cells.

### Downregulation of SLCO4C1 inhibited the apoptosis in HCC cells and affected the progression of EMT

**Figure 11:**
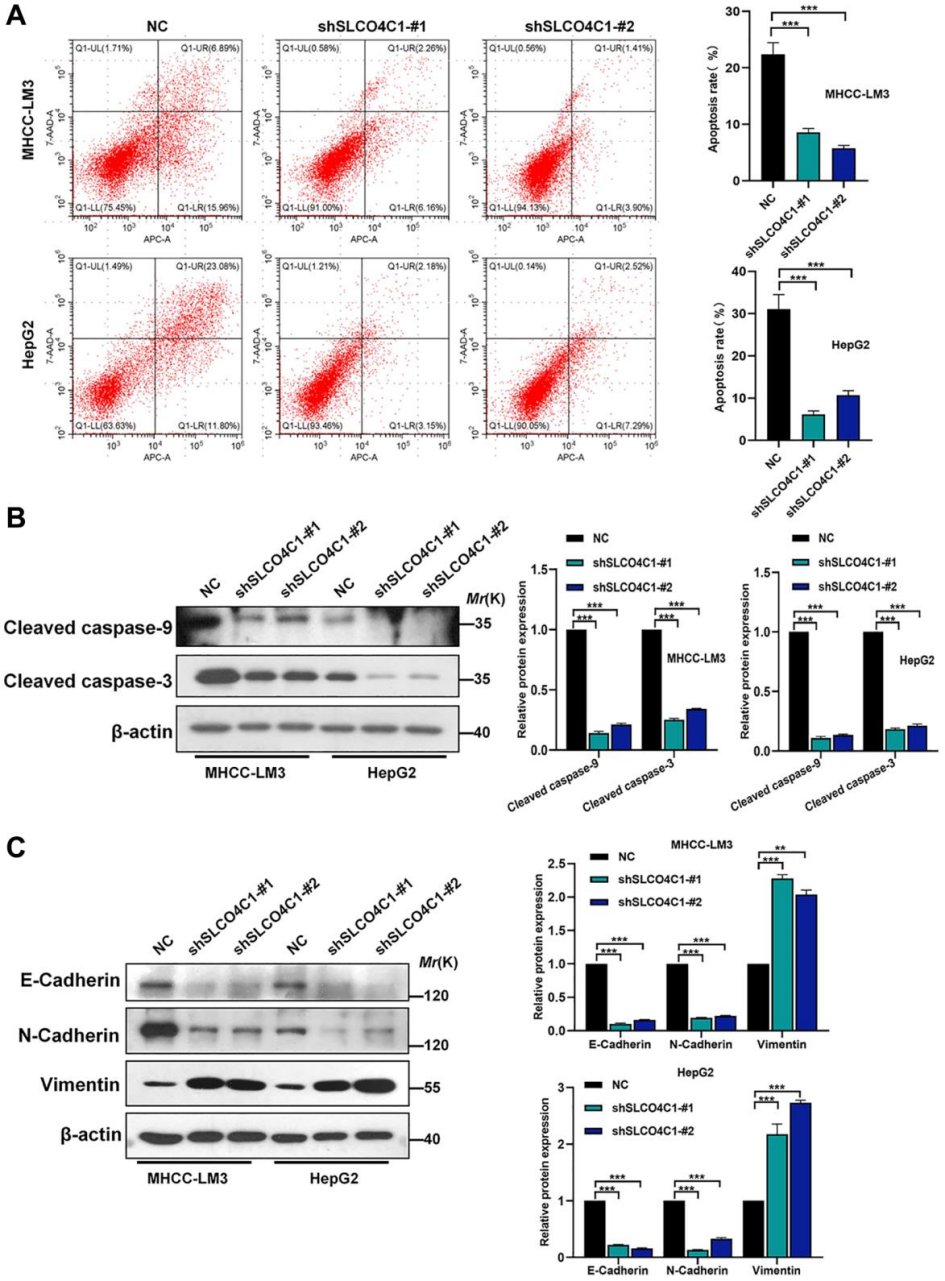
**Effect of SLCO4C1 knockdown on apoptosis in HCC cells.** (**A**) Flow cytometry analysis of apoptosis in HepG2 and MHCC-LM3 cells after SLCO4C1 knockdown. (**B**) Western blotting for the expression of apoptosis-related protein cleavage caspase-3/9 in SLCO4C1 knockdown HepG2 and MHCC-LM3 cells. (**C**) Expression of EMT markers in SLCO4C1 knockdown HepG2 and MHCC-LM3 cells by Western blotting. ^*^P < 0.05, ^**^P < 0.01, ^***^P < 0.001.

The rate of apoptosis detected in the SLCO4C1 knockdown group was reduced, indicating that SLCO4C1 knockdown inhibited the apoptosis of HCC cells (Figure 11A). In the SLCO4C1 knockdown group, decreased expression of the apoptosis-related protein caspase-3/9 was detected (Figure 11B). The metastatic progression of the tumor is tightly associated with EMT. We further found that E-cadherin and N-cadherin expression were significantly reduced, while vimentin expression was significantly upregulated in the SLCO4C1 knockdown group (Figure 11C). This suggests that reduced expression of SLCO4C1 can promote EMT progression in HCC cells.

## DISCUSSION

Due to their mild symptoms and rapid progression, the prognosis of HCC patients is generally poor [26, 27]. Mortality rate in HCC has largely not improved in the past decade [28]. The prognostic effect of traditional treatments is not satisfactory. In recent years, the research on HCC has been continuously deepened, including many new advances in etiology, prevention, diagnosis and treatment. The occurrence of HCC was associated with abnormal hepatocyte metabolism, which affects the behavior of HCC cells [29]. The investigators are continuing to explore the precise treatment of HCC. These therapeutic approaches could target specific molecules or signaling pathways in HCC cells to achieve control and treatment of HCC. Targeting certain immune checkpoints has improved the prognosis in advanced HCC [30]. However, there is a pressing need for predictive biomarkers to guide treatment selection. Studies based on the biological characteristics of molecular markers will provide more treatment options for HCC patients and improve the treatment efficacy and quality of life.

To further elucidate the pathogenesis and therapeutic targets of HCC, targeted therapy and immunotherapy have become novel options for HCC patients. The combination of targeting and immunity has achieved good results. This method can effectively fight the tumor, and has the advantages of less clinical side effects, low clinical recurrence rate and high degree of tumor remission [31, 32]. Recent research on the anoikis has also made good progress. Anoikis recovery was seen as an effective direction for antitumor therapy [33, 34]. A study in lung cancer showed that GDH1 promoted anoikis resistance and metastatic of tumors through CamKK2 activation [35]. CPT1A promoted metastasis in colorectal cancer cells by inhibiting anoikis. The PRMT5/MTHFD1 axis promotes tumor resistance and accelerates its metastasis in oesophageal squamous cell carcinoma [36]. These studies suggest that anoikis resistance was a key factor in cancer initiation and metastasis in tumors.

Thus, our study combines the analysis between HCC samples from 3 different databases and anoikis genes. We used clustering to classify all samples into two types (A and B), according to the anoikis gene. We found that the prognosis of subtypeB was obvious due to subtype A, and patients with subtype B had a greater survival advantage. To find DEGs between the two subtypes, we identified the gene modules most closely connected to anoikis using WGCNA and identified 491 genes. To explore the functional role of DEGs in different HCC subtypes, we developed a quantitative anoikis risk model. We found that the high-risk subtypes had a shorter OS.

Immunotherapy is a promising direction among the numerous treatments for HCC. CD8T cells and activated NK cells are considered to be the roles in immune cells involved in clear tumor cells [37]. CTLA4 is a common immune checkpoint, which is a protein receptor of T cells, and T cells are a type of leukocytes in the immune system. It functions as an immune checkpoint inhibitor by regulating the activation of T cells [38]. When activated, T cells were able to recognize and attack foreign substances, such as viruses or cancer cells. This makes CTLA4 an immunotherapy target for various autoimmune diseases and cancers, and blocking the CTLA4 pathway contributes to enhancing the immune response and attacking cancer cells. Ipilimumab as a CTLA4 blocker, it has been applied to the treatment of advanced melanoma [39, 40]. HCC responds differently to different drugs, which makes it difficult to assess the benefits of the drugs. The anoikis model we established could also help guide HCC patients in choosing appropriate therapeutic agents.

The 12 risk genes in the anoikis model play their respective roles in tumors. FI27 acts as an immune response in the immune system. ATF3 regulates the biological behavior of human squamous cell carcinoma through the downregulation of IFI27 [41]. IFI27 mRNA Promote angiogenesis in ESCC through exosomal miR-21-5p/CXCL10 [42]. NQO1 promoted the aggressive phenotype of in HCC through amplification of ERK-NRF 2 signaling [43]. RAB 26 played an inhibitory role in breast cancer, but it promoted cancer progression in lung cancer. Similarly, SLCO4C1 acting as an oncogene in endometrial cancer but as cancer suppression in head and neck cancer [18]. Our study identified a low expression of SLCO4C1 in HCC and could inhibit HCC.

Reducing SLCO4C1 expression significantly increased the proliferation viability, invasiveness and migratory ability of HCC cells. Meanwhile, the low expression of SLCO4C1 can also reduce the apoptosis in HCC cells. It can be determined that SLCO4C1 promotes the malignant phenotype of HCC cells. However, the low expression of SLCO4C1 inhibited the biological behavior of endometrial cancer cells and could promote the apoptosis of their tumor cells [18]. Our study elucidates the heterogeneity of SLCO4C1 in HCC tumor progression.

Members of the solute carrier family are associated with a variety of neoplastic diseases. Upreregulation of SLC7A11 suppresses ferroptosis through cystine and promotes the growth of tumor cells [44]. Cells overexpressing SLC7A11 under glucose starvation conditions induced disulfide disassembly between intracellular actin cytoskeletal proteins, causing the outcome of cell death [45]. SLC25A5 mediated the MAPK signaling pathway to inhibit the malignant behavior in colon cancer cells [46]. SOCS2 promoted the progression of ferroptosis in HCC cells by accelerating SLC7A11 degradation [47]. Previous studies have shown a role of SLCO4C1 in reducing renal inflammation and lowering blood pressure. The biological role of SLCO4C1 in HCC was first reported in this study, and the decreased expression of SLCO4C1 promoted the malignant action phenotype of HCC cells. The EMT process is generally recognized as an important event in the spread of cancer cells. Experiments confirmed that decreased SLCO4C1 expression resulted in decreased e-cadherin and n-cadherin expression and increased vimentin expression.

Our study classified HCC patients into different anoikis subtypes and identified key prognostic genes according to the DEGs between subtypes. An anoikis risk prognostic model and nomogram were constructed to predict OS. The feature model of anoikis has special accuracy and utility in predicting OS, assessing immune infiltration and drug sensitivity analysis. Our experiments provided the first evidence that SLCO4C1 suppresses HCC progression and demonstrated a mechanism of action. Meanwhile, the progression of apoptosis and EMT in HCC cells were also affected by the expression of SLCO4C1. In conclusion, this study provides a powerful predictive means for improving survival and provides new individualized management and new biological targets for the treatment of HCC.

## CONCLUSIONS

In conclusion, we developed a HCC classification approach for anoikis features and constructed new prognostic models. The new classification shows good potential in clinical prognosis and immunological features. Moreover, we successfully identified and validated the tumor-suppressive role of SLCO4C1 in HCC, which holds great potential for improving the diagnosis and treatment of HCC patients.

## Supplementary Materials

Supplementary Figure 1

Supplementary Table 1
