# Supplementary material for: Identification and validation of SLCO4C1 as a biological marker in hepatocellular carcinoma based on anoikis classification features
Source: Aging (Albany NY). 2024 Jan 15;16(2):1440–62. doi: 10.18632/aging.205438 (PMC10866452; doi:10.18632/aging.205438)
Supplement: Supplementary Figure 1 [file aging-16-205438-s001.pdf]

SUPPLEMENTARY FIGURE

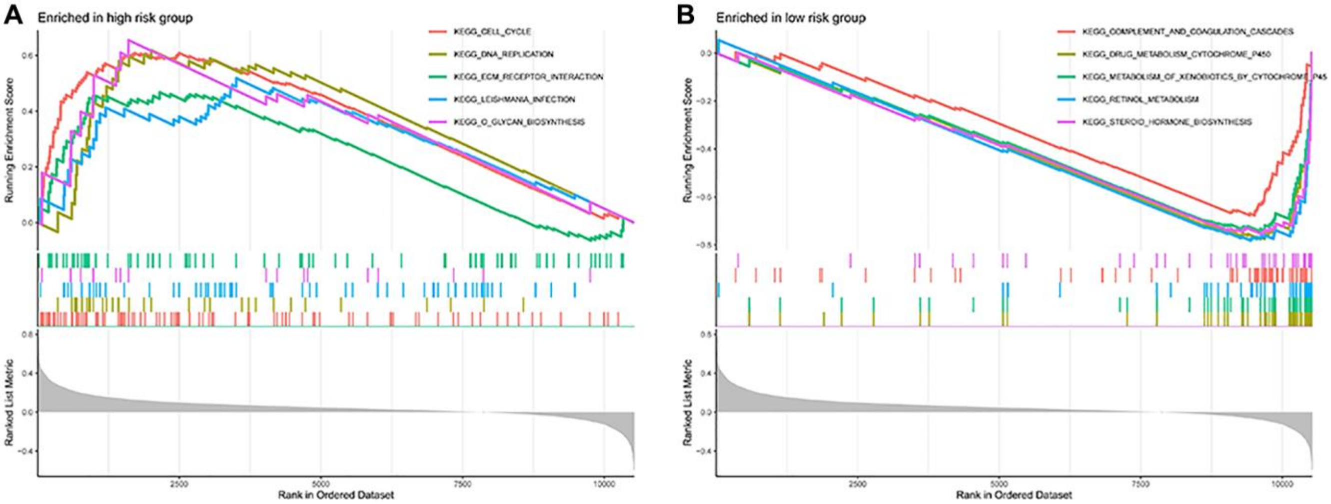

Supplementary Figure 1. (**A**, **B**) Enrichment map for gene collection enrichment analysis of high-risk and low-risk groups.
