# Supplementary material for: Identification and validation of SLCO4C1 as a biological marker in hepatocellular carcinoma based on anoikis classification features
Source: Aging (Albany NY). 2024 Jan 15;16(2):1440–62. doi: 10.18632/aging.205438 (PMC10866452; doi:10.18632/aging.205438)
Supplement: Supplementary Table 1 [file aging-16-205438-s002.docx]

**Supplementary Table 1. Gene files resulting from the univariate Cox analysis.**

| **id** | **HR** | **HR.95L** | **HR.95H** | **pvalue** |
| --- | --- | --- | --- | --- |
| DUSP10 | 0.764400783 | 0.667465006 | 0.875414517 | 0.00010313 |
| TRPM8 | 0.834633596 | 0.745062721 | 0.934972614 | 0.001803556 |
| USH1C | 1.085726853 | 1.013855527 | 1.162693073 | 0.018585244 |
| CCL25 | 0.907774693 | 0.835715849 | 0.986046745 | 0.021850878 |
| PPP1R3C | 1.11396851 | 1.013733312 | 1.224114692 | 0.024865059 |
| SPTBN2 | 0.827722962 | 0.733654187 | 0.933853189 | 0.002127754 |
| LOX | 1.301230246 | 1.171696391 | 1.445084379 | 8.58E-07 |
| SREBF1 | 1.134290654 | 1.006938609 | 1.277749483 | 0.038101473 |
| FXYD3 | 1.093642153 | 1.014595092 | 1.178847767 | 0.01936143 |
| FAS | 0.794107544 | 0.69212266 | 0.911119991 | 0.001011926 |
| CPM | 1.144580319 | 1.000136274 | 1.309885603 | 0.049769034 |
| GCK | 0.896157471 | 0.814100498 | 0.986485349 | 0.02524252 |
| KRT8 | 1.178627384 | 1.033533589 | 1.344090337 | 0.014203639 |
| STC1 | 1.243029701 | 1.117262981 | 1.382953579 | 6.41E-05 |
| LIME1 | 0.843216608 | 0.741374477 | 0.959048727 | 0.009413823 |
| CDKN1C | 1.28166011 | 1.159439181 | 1.416764816 | 1.22E-06 |
| SLC1A7 | 1.131688701 | 1.023179533 | 1.251705369 | 0.016148477 |
| BOK | 0.807240829 | 0.710395244 | 0.917289019 | 0.001023551 |
| LEFTY1 | 1.208503731 | 1.087091725 | 1.343475657 | 0.000455234 |
| TXNIP | 0.876996182 | 0.799157956 | 0.962415875 | 0.00564361 |
| GDF15 | 1.11135929 | 1.020299825 | 1.210545608 | 0.015489964 |
| S100A14 | 1.067648836 | 1.005505249 | 1.133633105 | 0.032403359 |
| TSPAN7 | 1.134370666 | 1.008034433 | 1.276540528 | 0.036367335 |
| KEL | 1.164327083 | 1.035064353 | 1.30973263 | 0.011278233 |
| MPPED1 | 0.853215189 | 0.760683984 | 0.957002085 | 0.006721189 |
| CYP17A1 | 0.944562992 | 0.892560509 | 0.999595252 | 0.048384514 |
| LARP6 | 1.181456029 | 1.052398879 | 1.326339637 | 0.004723433 |
| SERPINE1 | 1.178090287 | 1.097088014 | 1.265073272 | 6.50E-06 |
| TMEM45A | 1.12157653 | 1.045948625 | 1.202672754 | 0.001276443 |
| BDH2 | 0.874326963 | 0.769859648 | 0.992970134 | 0.038581111 |
| APOL5 | 0.799576981 | 0.67977398 | 0.940493998 | 0.006918638 |
| BMP2 | 1.133056732 | 1.017131985 | 1.262193674 | 0.023302851 |
| ADAMTSL3 | 0.862205104 | 0.760921664 | 0.976970004 | 0.020050603 |
| CLDN18 | 1.165975651 | 1.048780039 | 1.296267252 | 0.004494601 |
| TREH | 0.816179501 | 0.708863833 | 0.939741804 | 0.004741989 |
| COPZ2 | 0.84133376 | 0.741866436 | 0.954137377 | 0.007117499 |
| DDT | 0.857224931 | 0.752194616 | 0.976920822 | 0.020882662 |
| G0S2 | 0.933148603 | 0.87317014 | 0.997247014 | 0.041221445 |
| SLC2A10 | 0.89636171 | 0.815386567 | 0.985378407 | 0.023520432 |
| FTL | 1.210198994 | 1.051265896 | 1.393160009 | 0.0079084 |
| OASL | 0.826034569 | 0.745733714 | 0.914982245 | 0.000249489 |
| GPC1 | 1.1328343 | 1.016959309 | 1.26191239 | 0.023486633 |
| COL4A5 | 1.130189718 | 1.030568383 | 1.239441089 | 0.009335306 |
| MAFF | 1.273477888 | 1.138069818 | 1.424996873 | 2.50E-05 |
| SRI | 1.28915511 | 1.125432731 | 1.476695009 | 0.000247145 |
| PER3 | 0.824347485 | 0.717924165 | 0.946546737 | 0.006164572 |
| KRT20 | 1.150010311 | 1.072166686 | 1.233505698 | 9.29E-05 |
| TFF1 | 1.0721146 | 1.004272499 | 1.144539671 | 0.036816539 |
| AQP8 | 0.845300636 | 0.754389367 | 0.947167599 | 0.00379228 |
| MATN3 | 1.196177604 | 1.078444437 | 1.326763634 | 0.000702684 |
| B4GALNT1 | 1.25359375 | 1.111182784 | 1.414256333 | 0.00023929 |
| P4HA2 | 1.167923267 | 1.028592475 | 1.326127492 | 0.016624348 |
| ADM | 1.306390691 | 1.189921613 | 1.434259718 | 2.03E-08 |
| IGFALS | 0.851321206 | 0.78367162 | 0.924810567 | 0.000138822 |
| RPL9 | 1.122707603 | 1.033864411 | 1.219185368 | 0.005927912 |
| ACTG2 | 1.133124473 | 1.016354865 | 1.26330981 | 0.024302004 |
| NTS | 1.090669966 | 1.044978538 | 1.138359242 | 7.04E-05 |
| MAPK12 | 1.224604826 | 1.083431198 | 1.384173709 | 0.001186006 |
| SPINK1 | 1.057174457 | 1.01901827 | 1.096759366 | 0.003032209 |
| BAMBI | 1.201974092 | 1.105396588 | 1.306989486 | 1.67E-05 |
| PAEP | 1.119659748 | 1.051142835 | 1.192642816 | 0.000451343 |
| FCGBP | 1.275408483 | 1.153887296 | 1.409727627 | 1.92E-06 |
| MAN1C1 | 0.863007226 | 0.756168658 | 0.984940944 | 0.028889263 |
| TFF2 | 1.127177543 | 1.057934047 | 1.200953139 | 0.000214729 |
| SPARCL1 | 0.897408279 | 0.832323095 | 0.96758293 | 0.004834938 |
| TMPRSS3 | 1.102825394 | 1.016244074 | 1.196783214 | 0.01896431 |
| IFIT1 | 0.820745928 | 0.748334478 | 0.900164162 | 2.77E-05 |
| IFI27 | 0.910728278 | 0.859686179 | 0.964800897 | 0.001484763 |
| SQSTM1 | 1.204659355 | 1.062348686 | 1.366033752 | 0.003697052 |
| CXCL5 | 1.121322678 | 1.044289384 | 1.204038428 | 0.00161391 |
| TUSC3 | 1.100605841 | 1.008377292 | 1.201269829 | 0.031809926 |
| CTSE | 1.132065323 | 1.051085003 | 1.219284731 | 0.001054188 |
| FKBP11 | 1.201392267 | 1.056421298 | 1.366257365 | 0.005165554 |
| CYB561 | 1.131654249 | 1.011358033 | 1.266259127 | 0.031011435 |
| SLC2A6 | 1.233133783 | 1.107792675 | 1.372656601 | 0.000127211 |
| GPR35 | 1.116051613 | 1.006093796 | 1.238026918 | 0.038008312 |
| HSPA6 | 1.164744616 | 1.072108005 | 1.265385591 | 0.000310221 |
| ACSL6 | 0.762014654 | 0.658311776 | 0.882053692 | 0.000271105 |
| SLC7A10 | 1.13721928 | 1.03049834 | 1.254992503 | 0.010543087 |
| UPK3A | 1.07979605 | 1.02083033 | 1.142167778 | 0.00737277 |
| S100A13 | 1.157727158 | 1.037146527 | 1.292326723 | 0.009056233 |
| FBXO2 | 0.915963456 | 0.842083128 | 0.996325688 | 0.040780538 |
| ASPHD1 | 1.186949564 | 1.089232632 | 1.29343285 | 9.23E-05 |
| SPP1 | 1.13346409 | 1.089287556 | 1.179432223 | 6.56E-10 |
| NR2F1 | 1.158692351 | 1.034602277 | 1.297665773 | 0.010817132 |
| S100A8 | 1.103286225 | 1.020511504 | 1.192774888 | 0.013502712 |
| EPO | 1.321317787 | 1.223520043 | 1.426932648 | 1.23E-12 |
| AGPAT2 | 1.164331003 | 1.02862716 | 1.317937866 | 0.016111672 |
| UGCG | 1.171768247 | 1.033171811 | 1.328956918 | 0.013584412 |
| TCEA2 | 0.848459469 | 0.740705458 | 0.971888979 | 0.017719115 |
| ART4 | 0.853688784 | 0.765534411 | 0.951994488 | 0.004446245 |
| STEAP1 | 1.133039547 | 1.051971286 | 1.220355186 | 0.000975165 |
| PROM1 | 1.132814006 | 1.039115708 | 1.23496119 | 0.004639719 |
| GUCY2C | 1.132380864 | 1.032164223 | 1.24232791 | 0.008549469 |
| RND1 | 0.883174551 | 0.800736007 | 0.974100429 | 0.012961804 |
| GFRA1 | 0.812591924 | 0.729643123 | 0.904970683 | 0.000158367 |
| CD5L | 0.854403615 | 0.788622325 | 0.925671916 | 0.000118382 |
| SEMA6A | 1.213633074 | 1.092040126 | 1.348764759 | 0.000324886 |
| SULT4A1 | 0.830469426 | 0.728686135 | 0.946469864 | 0.005358097 |
| RRS1 | 1.23007555 | 1.076306625 | 1.405813012 | 0.00237176 |
| TMPRSS2 | 0.869961026 | 0.771548843 | 0.980925827 | 0.022942808 |
| MSC | 1.133368941 | 1.05337081 | 1.219442522 | 0.000801787 |
| NPTX2 | 1.084298064 | 1.019514739 | 1.153197933 | 0.010028798 |
| TSPAN13 | 1.160623073 | 1.081068652 | 1.2460318 | 3.93E-05 |
| ABCC4 | 1.158332918 | 1.041227882 | 1.288608548 | 0.006873571 |
| SLC39A4 | 1.139529193 | 1.058528085 | 1.226728701 | 0.000516852 |
| RTP4 | 0.780020382 | 0.693993259 | 0.876711394 | 3.09E-05 |
| SLCO4C1 | 1.140618204 | 1.025702291 | 1.268408874 | 0.015167771 |
| ANXA2P2 | 1.442101095 | 1.263156494 | 1.646395818 | 6.10E-08 |
| EPHB6 | 1.199988523 | 1.067161651 | 1.349348015 | 0.002319071 |
| ENTPD2 | 1.194425878 | 1.063688868 | 1.341231652 | 0.002665578 |
| RAB26 | 0.817674148 | 0.726611509 | 0.920149219 | 0.000833615 |
| AMIGO2 | 1.185895189 | 1.06277137 | 1.323283107 | 0.002299901 |
| KRT17 | 1.162003623 | 1.089958269 | 1.238811117 | 4.27E-06 |
| STC2 | 1.386704722 | 1.248706457 | 1.539953585 | 9.78E-10 |
| SGCB | 1.272050106 | 1.127282681 | 1.435408793 | 9.48E-05 |
| ALDH1B1 | 0.836010969 | 0.741057935 | 0.943130499 | 0.003593434 |
| GULP1 | 1.233435476 | 1.090011834 | 1.395730787 | 0.000879379 |
| SULT1A1 | 0.862731251 | 0.777912559 | 0.956798039 | 0.005168194 |
| HKDC1 | 1.09232956 | 1.004997807 | 1.18725022 | 0.037780705 |
| DHRS2 | 0.924329714 | 0.873276929 | 0.978367105 | 0.006639275 |
| NDUFA4L2 | 1.251029631 | 1.140285547 | 1.372529137 | 2.18E-06 |
| TES | 1.192847182 | 1.059277846 | 1.343258904 | 0.003609704 |
| GGT1 | 1.092872387 | 1.012257456 | 1.179907391 | 0.023111827 |
| RAC3 | 1.170615624 | 1.03874401 | 1.319228729 | 0.009785262 |
| GUCA2A | 1.141999782 | 1.032105003 | 1.263595756 | 0.010108534 |
| ALPK3 | 1.159947286 | 1.032220744 | 1.303478654 | 0.012675943 |
| CLDN4 | 1.086845866 | 1.02617828 | 1.151100115 | 0.004486641 |
| POF1B | 1.23138138 | 1.119056571 | 1.354980743 | 2.00E-05 |
| TUBB2A | 1.128742838 | 1.008303257 | 1.263568659 | 0.035413625 |
| PRAME | 1.137492326 | 1.0626367 | 1.217621028 | 0.00020793 |
| PLEK2 | 1.08499928 | 1.00435125 | 1.172123236 | 0.038439149 |
| HOXA5 | 1.143883367 | 1.008051071 | 1.298018717 | 0.037133392 |
| FOLR1 | 1.160255709 | 1.049531431 | 1.282661262 | 0.003676251 |
| TNFRSF11B | 1.093813133 | 1.00181205 | 1.194263106 | 0.045462428 |
| GLRB | 1.173078137 | 1.042116337 | 1.320497787 | 0.008217631 |
| QPCT | 1.147013479 | 1.041918308 | 1.262709284 | 0.005150394 |
| KDELR3 | 1.24475849 | 1.130897818 | 1.370082844 | 7.70E-06 |
| TSC22D3 | 0.847440988 | 0.763506679 | 0.940602419 | 0.001866634 |
| IL1RN | 0.897142565 | 0.805089404 | 0.999720997 | 0.049412414 |
| PTGES | 1.154997172 | 1.057363662 | 1.261645842 | 0.001384827 |
| APOL6 | 0.728328585 | 0.634498787 | 0.83603395 | 6.64E-06 |
| RAB32 | 1.436015083 | 1.238986158 | 1.664376399 | 1.54E-06 |
| SSTR2 | 1.141983072 | 1.021633726 | 1.276509675 | 0.019457294 |
| NRCAM | 1.174252817 | 1.074640648 | 1.283098382 | 0.000382915 |
| EBP | 1.157011047 | 1.006432249 | 1.330118908 | 0.040355311 |
| FLRT3 | 1.113518001 | 1.007353002 | 1.230871736 | 0.03544228 |
| ETV4 | 1.157400158 | 1.071065976 | 1.250693379 | 0.000219252 |
| IL1RAP | 1.117288099 | 1.000942746 | 1.247156943 | 0.048068813 |
| VNN2 | 1.171594468 | 1.093184867 | 1.255628063 | 7.43E-06 |
| EVPL | 1.162090737 | 1.027931771 | 1.31375926 | 0.016389754 |
| PERP | 1.169035928 | 1.036391801 | 1.318656707 | 0.011031866 |
| SEC24C | 1.14785491 | 1.005323935 | 1.310593381 | 0.041504222 |
| RCN1 | 1.13939425 | 1.006758788 | 1.289503775 | 0.038768001 |
| C3orf14 | 1.150575901 | 1.026347563 | 1.289840744 | 0.016124658 |
| ELF3 | 1.152303724 | 1.040769404 | 1.275790649 | 0.006346894 |
| AP1M2 | 1.100582057 | 1.038376654 | 1.16651396 | 0.001244017 |
| SOAT2 | 1.089655878 | 1.011758845 | 1.173550335 | 0.023275919 |
| PPARG | 1.321893071 | 1.166932887 | 1.497430839 | 1.15E-05 |
| CA12 | 1.164099436 | 1.078699072 | 1.256260928 | 9.28E-05 |
| GSTM2 | 0.846798208 | 0.737708195 | 0.972020115 | 0.018114533 |
| ADRA2C | 1.117039656 | 1.016194288 | 1.227892744 | 0.021863261 |
| DDIT4 | 1.210563854 | 1.108073521 | 1.322533944 | 2.30E-05 |
| EFHD1 | 0.890695257 | 0.81203371 | 0.976976734 | 0.014138948 |
| IGFBP3 | 1.18117173 | 1.085863948 | 1.284844808 | 0.000104869 |
| MSI1 | 1.110963182 | 1.010060405 | 1.22194592 | 0.030310122 |
| CCDC28B | 1.163858524 | 1.022146167 | 1.325218161 | 0.021985007 |
| SLC16A4 | 0.81835529 | 0.70779414 | 0.946186671 | 0.006790986 |
| S100P | 1.075011739 | 1.034374535 | 1.117245447 | 0.000234191 |
| ACTL8 | 1.137484515 | 1.03021969 | 1.255917581 | 0.010800157 |
| SPON2 | 1.147178128 | 1.024408898 | 1.28466051 | 0.017428193 |
| NQO1 | 1.101475358 | 1.053524698 | 1.151608468 | 2.08E-05 |
| ACSL4 | 1.068504555 | 1.007875091 | 1.13278123 | 0.026205445 |
| NXPH4 | 1.163600848 | 1.071389264 | 1.263748834 | 0.000322018 |
| WNT5B | 0.869705468 | 0.76236808 | 0.992155392 | 0.037788117 |
| SORT1 | 1.198213114 | 1.047472017 | 1.370647276 | 0.008387455 |
| ARG2 | 1.145023073 | 1.02341488 | 1.281081469 | 0.018079601 |
| RPS6KA3 | 1.175964812 | 1.025035395 | 1.349117549 | 0.020734388 |
| ME1 | 1.131903018 | 1.040201943 | 1.231688184 | 0.004048687 |
| MYCN | 1.253503878 | 1.116452774 | 1.407378807 | 0.000130968 |
| HSPA1B | 1.13598374 | 1.040898861 | 1.239754511 | 0.004253505 |
| RAMP1 | 0.878260362 | 0.817888839 | 0.943088139 | 0.000353481 |
| MAGEH1 | 1.119619893 | 1.000043974 | 1.253493582 | 0.04991087 |
| GSTM1 | 0.936249899 | 0.888937277 | 0.986080679 | 0.012782904 |
| SCPEP1 | 1.144137388 | 1.034164894 | 1.265804293 | 0.009014238 |
| BBOX1 | 0.902514309 | 0.835974748 | 0.974350098 | 0.008666115 |
| PHLDA3 | 0.892093166 | 0.807791118 | 0.985193077 | 0.024164534 |
| FLNC | 1.142101307 | 1.055650162 | 1.235632259 | 0.000938049 |
| NEDD9 | 1.245762053 | 1.10084407 | 1.409757417 | 0.000496522 |
| PPP1R1A | 0.917364503 | 0.863099281 | 0.975041514 | 0.005564427 |
| ZNF83 | 1.096852381 | 1.003068077 | 1.199405279 | 0.04264828 |
| CLCF1 | 1.173913424 | 1.026999136 | 1.341844096 | 0.018748142 |
